# Assessment of vaccination rates and motivation among transplant patients using vaccination cards and interviews

**DOI:** 10.1038/s41598-025-01491-4

**Published:** 2025-05-15

**Authors:** Yvonne Remane, Vincent Christoph Klaus, Katrin Heinitz, Donald Ranft, Jan Kowald, Adam Herber, Hans-Michael Tautenhahn, Thilo Bertsche, Svitlana Ziganshyna

**Affiliations:** 1 Pharmacy, University of Leipzig Medical Center and Leipzig University Medical Faculty, Leipzig, Germany; 2 Drug Safety Center, Leipzig University and University of Leipzig Medical Center, Leipzig, Germany; 3 Medical Department III, Division of Nephrology, University of Leipzig Medical Center, Leipzig, Germany; 4 Medical Department II, Division of Hepatology, University of Leipzig Medical Center, Leipzig, Germany; 5 Department of Visceral, Transplantation, Thoracic and Vascular Surgery, University of Leipzig Medical Center, Leipzig, Germany; 6 Clinical Pharmacy, Institute of Pharmacy, Medical Faculty, Leipzig University, Leipzig, Germany; 7 Organ Donation Coordinator Unit, University of Leipzig Medical Center, Leipzig, Germany

**Keywords:** Vaccination hesitancy, Immunization coverage, Transplant recipients, Immunization schedule, Vaccine-preventable diseases, Vaccine compliance, Risk factors, Epidemiology

## Abstract

Transplant patients are at an elevated risk for infections. Therefore, infection prevention plays a pivotal role in their care. Two different approaches were chosen to determine their vaccination status and motivation. The vaccination rate was determined by analyzing the patients’ vaccination cards based on German vaccination recommendations. The vaccinations were categorized into standard, indicated, and risk-dependent vaccinations. The vaccination motivation was determined through a semi-structured interview using a self-developed questionnaire as an interview guide. Both parts were analyzed separately. A total of 126 patients were included in the study. In 115 (91.3%) patients, vaccination cards were available, 64.3% had complete standard and 0.9% indicated vaccinations. In the risk-dependent vaccinations category, 49.6% of participants had at least one of the additional vaccinations. The vaccination rate against hepatitis B was significantly higher in kidney than in liver transplant recipients (88.1% vs. 55.4%, Χ2 = 13.7, p < 0.001, n = 107). Vaccination confidence correlated significantly with willingness to vaccinate (r = 0.362, p < 0.001, n = 123). In conclusion, only a few transplant patients have complete vaccination protection. Further patient education is needed to increase patients’ confidence in vaccinations and to motivate them.

## Introduction

Infections are among the most critical risk factors for graft rejection and injury^1–3^. Lifelong immunosuppression is associated with an increased risk of infection, a more severe clinical outcome, and increased morbidity and mortality^4,5^. Infectious diseases such as coronavirus (COVID-19), influenza, measles, pneumococci, or varicella zoster have been shown to cause graft rejection, hospitalization, or graft injury^2,6,7^. Vaccination is the most effective protection against vaccine-preventable diseases^8^. Therefore, transplant patients should be adequately immunized. Even before transplantation, the effectiveness of the vaccine may be reduced. Based on the mostly chronic primary disease with organ failure and its effects on the metabolism, there could be a functional impairment of the immune system^9,10^. The pharmacological suppression of the immune system also affects the efficacy of vaccinations, impeding the desired humoral and cellular immune response to the vaccine antigens. This phenomenon is associated with a reduced seroconversion rate in organ transplant recipients when vaccinations are administered post-transplantation. According to the conventional vaccination schedule, the serological evidence for immunogenicity is often absent^11–14^. The German Standing Committee on Vaccination (STIKO) at the Robert Koch Institute (RKI) has developed and published a vaccination recommendation for transplant patients^15^. The STIKO recommended the administration with inactivated vaccines up to two weeks before the scheduled organ transplantation. For live vaccines, the recommended period is four weeks before transplantation. In the case of persistent immunosuppression, live vaccines are contraindicated in general. Therefore, they must be administered before transplantation. Despite the potential for reduced immune response and immunosuppression, it is possible to administer inactivated vaccines after transplantation. However, it is recommended that these vaccines be administered after the induction phase of immunosuppression, which usually lasts three to six months. There may be vaccine-specific exceptions^15^. The Robert Koch Institute last published vaccination rates in 2022 in its annual report on vaccination monitoring. However, this report only shows the overall vaccination rates and provides no differentiation for vulnerable patient groups, such as transplant patients. According to these published data, the standard vaccination status of the Saxon population is at the national average. The vaccination rates for influenza and pneumococcal vaccinations for people with a vaccine-relevant primary disease, including transplant patients, is 34.1% in Saxony compared to 25.6% nationwide^16^. Despite the publication of STIKO recommendations tailored to the immunological needs of transplant patients, their actual implementation has not yet been systematically evaluated. This study therefore aimed to assess the vaccination status of organ transplant patients in a large transplant center in Saxony according to STIKO recommendations. In addition, patients’ willingness to be vaccinated was analyzed, and reasons for and barriers to vaccination motivation were evaluated.

## Materials and methods

### Setting and study design

This study was performed between February and April 2024 at the University Medical Center Leipzig, Transplant outpatient clinic. After receiving ethical approval from the responsible local ethics committee on January 30, 2024 (registration number 422/23-ek) patients were recruited following their scheduled appointments. The data received from the patients were entered into a database in an anonymized, aggregated form for further data processing. In addition to the vaccination status, the following data were recorded: age, sex, medical background, date of transplantation, time spent on the waiting list, reason for transplantation, and pre-existing illnesses. In addition, medical contraindications to vaccination were recorded. The patient’s vaccination card was the primary source for determining vaccination status. We recorded vaccinations, dates, and administering physicians. Therefore, we could identify the vaccinations that occurred before and after transplantation. If there is a discrepancy between the card and the records, the card is considered valid. Furthermore, any responses of 'do not remember’ were treated as indicating that the vaccination had not been received.

### Inclusion and exclusion criteria

Patients must be at least 18 years of age and proficient in German to be included in the study. The study included transplant patients only, who received care in our outpatient clinic. Furthermore, they must have undergone a successful organ transplantation. Due to the center’s focus on specific organ transplants, only liver, kidney, and pancreas transplants were included in the study. No further exclusion criteria were applied. All patients gave their written informed consent.

### Vaccination status determination

To evaluate the completeness of the vaccination program, the recommended vaccinations of the STIKO were split into three categories: standard, indicated, and occupational vaccinations. Furthermore, the specific recommendations of the STIKO regarding vaccinations for transplant patients were considered. The standard vaccination schedule includes vaccines against tetanus, diphtheria, and pertussis (Tdap), as well as against polio and measles, mumps, and rubella (MMR). According to the definition of the STIKO, all individuals born before 1970 are considered immune to MMR. The vaccination schedule recommended for transplant patients, due to their immunosuppression and heightened infection risk, includes vaccinations against hepatitis A (HepA) and B (HepB), against meningococcal serogroups A, C, W, Y (MenACWY) and B (MenB), pneumococcal disease (PCV), influenza, and the coronavirus (COVID-19). In the third category are those vaccinations that, while recommended as standard and indicated vaccines in general, depend on age and personal risk. These include vaccines against human papillomaviruses (HPV), Haemophilus influenzae type B (Hib), and against tick-borne encephalitis (TBE). Vaccination against Hib has only been recommended in Germany since 1990 for up to the age of five years. Therefore, only patients 38 years old or younger could have received this vaccine. Likewise, the HPV vaccination has been the recommended standard vaccination for both boys and girls since 2018. This resulted in the assumption that individuals under 24 should have received this vaccine as a standard vaccination. The recommended vaccination schedule for patients aged 50 years or older includes the herpes zoster virus (HZV) vaccine and, in cases of seronegative status, the varicella-zoster vaccine (VZV).

### Patient interview

We collected data on vaccination motivation and readiness through a semi-structured interview. A questionnaire was developed to guide the interview, which was conducted in the context of the study. The development was based on a previously tested and validated questionnaire on vaccine skepticism, vaccination motivation, and vaccine anxiety^17,18^. A pilot testing with five staff members was conducted to assess clarity and comprehension, using a Likert scale for evaluation.

If the vaccination card was unavailable or incomplete, the data were collected through patient statements during the interview. These statements were analyzed separately. We performed a semi-structured interview that included both closed-ended and open-ended questions. The closed-ended questions, which were asked precisely, allowed us to obtain comparable responses from the interviewees. The open-ended questions were designed to learn more about the background and motivations of the individuals^19^. The decision to be vaccinated is a matter of considerable emotionality and personal opinion. Consequently, it varies greatly from one individual to another. Semi-structured interviews allow further questioning to ensure that all relevant aspects are considered.

### Statistical analysis

The data were analyzed using IBM SPSS Statistics (Version 29, IBM, Armonk, USA). The descriptive presentation used absolute and relative frequencies of the individual vaccination rates. A distinction was made as to whether the data were provided by the vaccination card or from the interview with the participants. A Pearson chi-square test was used to investigate correlations between the type of transplantation and the vaccination rates. The effect size of the Pearson chi-square test was assessed using the correlation coefficient Kendall-Tau-b. In addition, a Spearman-Rho correlation analysis was performed to examine the relationships between the questionnaire items. To ascertain any discrepancies in vaccination status before and after transplantation, a Wilcox test was conducted. The analyzed items’ medians, the Mann–Whitney U-test’s rank sum, and the significance value p were used.

## Results

### Characteristics of participating patients

One hundred forty outpatient transplant recipients were screened from the 6th of February to the 28^th^ of March 2024, of which 126 could be included (53 (42.1%) female). The mean age of the patients was 58.7 years (SD 13.8). The median time since transplantation was 64 months, with 55.5% being liver, 38.1% kidney, and 3.2% kidney-pancreas transplant patients. A further 3.2% had a combined transplant, such as kidney and liver. Of all participants, 91.3% (n = 115/126) had a vaccination card. No cases of graft failure were observed during the period under review.

### Vaccination status analysis

#### Vaccination rates for patients with a vaccination card

Primary vaccination against Tdap was present in 87.0% (n = 100/115), of these, 85.0% (n = 85/100) received a booster within the last ten years. The vaccination rate for primary vaccination against polio was 80.0% (n = 92/115), of which 97.8% (n = 90/92) had a single booster vaccination in adulthood. Vaccination against HPV was available in 4.3% (n = 5/115). Since 2018, the HPV vaccination has been recommended as a standard vaccination for both boys and girls, assuming that individuals under 24 should have received this vaccination as a standard vaccination. In our study, the HPV vaccination rate for individuals younger than 24 was 100.0% (n = 5/5).

The vaccination rate for Hib was 7.0% (n = 8/115). Patients younger than 38 were vaccinated in 54.5% (n = 6/11). The vaccination rates for HepA and HepB were 36.5% (n = 42/115) and 68.7% (n = 79/115), respectively. Primary vaccination against PCV was given in 47.8% (n = 55/115). Within the last six years, a booster vaccination to complete primary vaccination was present in 48.3% (n = 14/29). The rates for the vaccination against MenACWY and MenB were 4.3% (n = 5/115) and 7.0% (n = 8/115). A primary vaccination against TBE was present in 34.8% (n = 40/115). A booster vaccination within the last five years and chronological indication were documented in 51.5% (n = 17/33). The vaccination rate against influenza for the 2023/2024 season was 49.6% (n = 57/115). For COVID-19, the primary vaccination rate, consisting of three antigen contacts, with at least one being a vaccination, was present in 91.3% (n = 105/115). A booster was administered in 25.7% (n = 27/105) in the 2023/2024 season. A summary of the results is shown in Table 1.

**Table 1:** Overview of Vaccination Rates for recommended Vaccinations according to STIKO and patient’s vaccination card (n = 115).

	Vaccination rates		
	Performed (n/%)	Not performed (n/%)	Started but not completed (n/%)
Tdap			
Primary vaccination (n = 115)	100/87%	15/13%	0/0%
Booster (n = 100)	85/85%	15/15%	0/0%
Polio			
Primary vaccination (n = 115)	92/80%	23/20%	0/0%
Booster (n = 92)	90/98%	2/2%	0/0%
HPV			
Overall (n = 115)	5/4%	109/95%	1/1%
HPV ≤ 23 years (n = 5)	5/100%	0/0%	0/0%
Hib			
Overall (n = 115)	8/7%	106/92%	1/1%
Hib ≤ 38 years (n = 11)	6/55%	4/36%	1/1%
HepA (n = 115)	42/37%	62/54%	11/10%
HepB (n = 115)	79/69%	29/25%	7/6%
PCV			
Primary vaccination (n = 115)	55/48%	48/42%	12/10%
Booster (n = 29)	14/48%	15/52%	0/0%
MenACWY (n = 115)	5/4%	104/90%	6/5%
Men B (n = 115)	8/7%	106/92%	1/2%
TBE			
Primary vaccination (n = 115)	40/35%	67/58%	8/7%
Booster (n = 33)	17/52%	16/49%	0/0%
Influenza 2023/2024 (n = 115)	57/50%	58/50%	0/0%
COVID-19			
Primary vaccination (n = 115)	105/91%	8/7.0%	2/2%
Booster 2023/2024 (n = 105)	27/26%	78/74.3%	0/0%

Recommendations for transplant patients include standard vaccination against tetanus, diphtheria, and pertussis (Tdap), Polio, as well as vaccinations against human papillomaviruses (HPV), Haemophilus influenzae type B (Hib), Hepatitis A/B (HepA/B), pneumococcal disease (PCV), meningococcal serogroups A, C, W, Y and B (MenACWY and B), tick-borne encephalitis (TBE), influenza, and coronavirus COVID-19.

The reported vaccination rates for primary vaccination or, overall, if only one vaccination is required, refer to the total number of 115 patients for whom a vaccination card was available. The vaccination rates for booster vaccines refer to the total number of patients who had a complete primary vaccination. For HPV and Hib vaccines, vaccination rates were reported for a specific age group because these vaccines were only standard vaccines for patients of a certain age.

Vaccination against HZV was received by 26.6% (n = 25/94) of patients over 50. Primary vaccination against MMR was completed in 20.9% (n = 24/115) but was not required in 67.8% (n = 78/115) of patients born in 1970 or earlier. For patients born after 1970, the vaccination rate against MMR was 64.9% (n = 24/37). Against VZV, 3.5% (n = 4/115) were vaccinated, and 50.4% (n = 58/115) had a medical history of chickenpox. Considering only those patients without VZV disease, the vaccination rate was 7.0% (n = 4/57). Details are shown in Table 2.

**Table 2:** Overview of vaccination rates against herpes zoster virus (HZV), measles, mumps, rubella (MMR), and varicella zoster virus (VZV) according to vaccination card and age (n = 115).

	Performed (n/%)	Not performed (n/%)	Started but not completed (n/%)
HZV ≥ 50 years	25/27%	68/72%	1/1%
MMR			
Overall*	24/21%	13/11%	0/0%
Born after 1970	24/65%	13/35%	0/0%
VZV			
Overall**	4/4%	53/46%	0/0%
Without known chickenpox infection	4/7%	53/93%	0/0%

One hundred fifteen patients were interviewed. The recommended vaccination schedule for patients aged 50 years or older includes the herpes zoster (HZV) vaccine and, in cases of seronegative status, the varicella vaccine (VZV). Patients born in 1970 or earlier did not require primary vaccination against MMR.

*Born before 1970 (n = 78; 68%).

**Chickenpox in Medical History (n = 58; 50%).

The data collected were analyzed based on standard, indication, and risk-dependent vaccinations. It was shown that 64.3% (n = 74/115) of the participants had a complete and up-to-date vaccination status for the standard vaccinations. In the area of indicated vaccinations, only 0.9% (n = 1/115) had complete and up-to-date vaccination protection against all recommended indicated vaccinations. In the risk-dependent vaccinations category, 49.6% (n = 57/115) of participants had at least one of the additional vaccinations (Table 3).

**Table 3:** Participants (n) with a vaccination card who have complete and up-to-date protection against all standard and indication vaccinations or at least one of the age- and/or risk-dependent vaccinations, analyzing 115 patients’ data.

	Standard vaccinations (n/%)	Indicated vaccinations (n/%)		Risk-dependent vaccinations (n/%)
Complete	74/64%	1/1%	At least one	57/50%
Incomplete	41/36%	114/99%	None	58/50%

#### Timing of vaccination

**Fig. 1:**
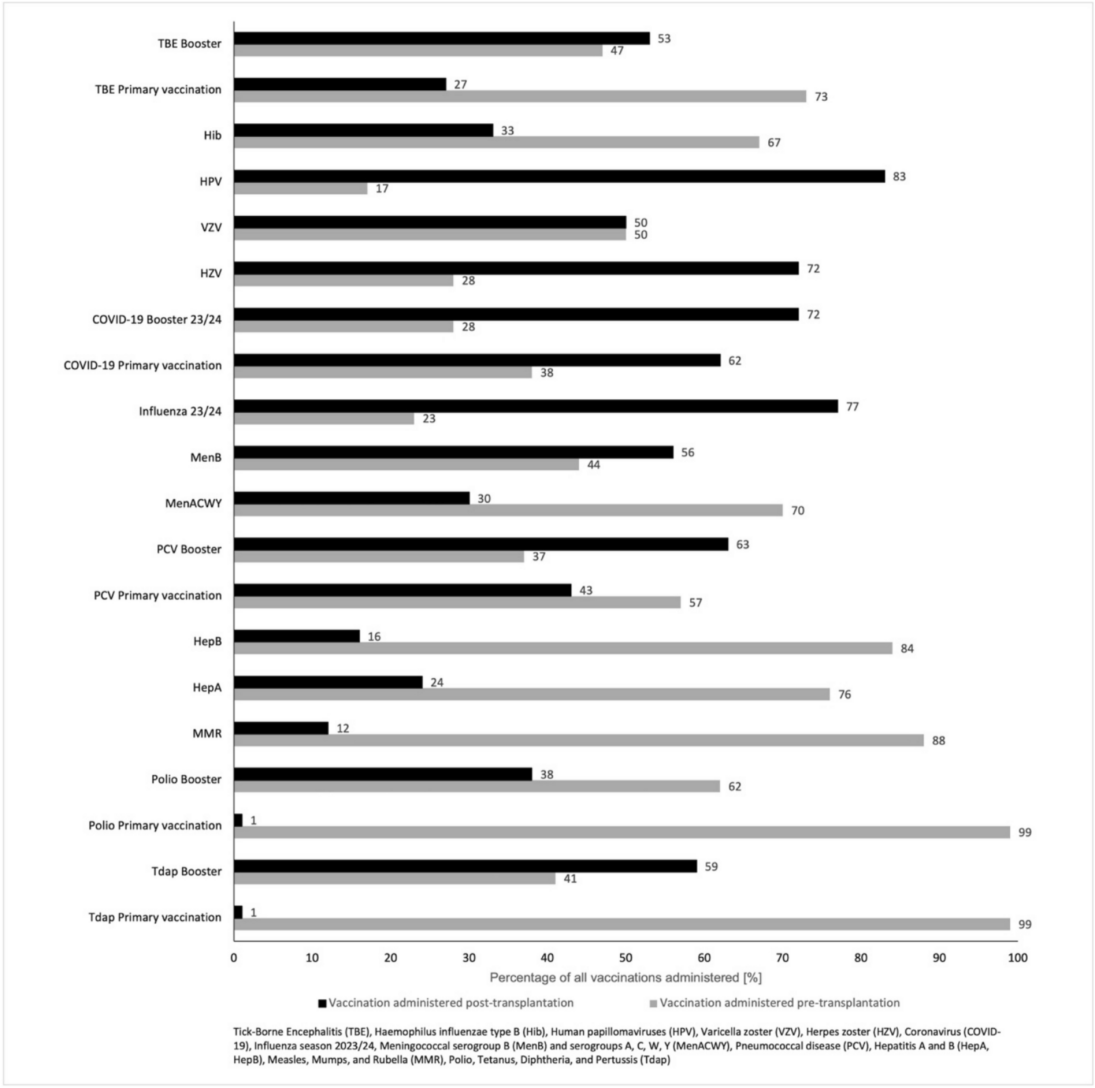
Overview of pre- and post-transplant vaccinations according to the participant’s vaccination card (n = 115). Based on the vaccination data documented in the vaccination card, the vaccinations pre- and post-transplantation were categorized and the percentage of vaccinations administered in the 115 patients was determined, including vaccination against tick-borne encephalitis (TBE), Haemophilus influenzae type B (Hib), human papillomaviruses (HPV), varicella vaccine (VZV), herpes zoster (HZV), coronavirus (COVID-19), influenza season 2023/24, meningococcal serogroup B (MenB) and serogroups A, C, W, Y (MenACWY), pneumococcal disease (PCV), hepatitis A and B (HepA + B), measles, mumps, and rubella (MMR), polio, tetanus, diphtheria, and pertussis (Tdap).

Based on the vaccination data documented in the vaccination card, the vaccinations were divided into pre- and post-transplant vaccinations. For each vaccination, the percentage of pre- and post-transplant vaccinations in relation to the total number of vaccinations administered was shown (Fig. 1). The proportion of live vaccines administered post-transplantation despite contraindications after risk assessment should be emphasized. As indicated on the vaccination card, this category includes three MMR vaccinations (3/24; 12.5%) and two VZV vaccinations (2/4; 50%).

#### Statistical sub-group analysis

Firstly, the vaccination rates between liver and kidney transplant patients were compared using cross-analysis tables and Pearson chi-square tests. The hepatitis B vaccination rate of kidney transplant patients (88.1%) was found to be significantly higher than that of liver transplant patients (55.4%) (Χ2 = 13.7; p < 0.001; n = 107). Secondly, the prevalence of immunity to varicella (either vaccination or a history of varicella-zoster virus (VZV) infection) was found to be significantly higher in the kidney transplant group (64.3% vs. 46.2%; Χ2 = 7.4; p < 0.05; n = 107). No other differences were observed in vaccination rates between the two groups.

Secondly, we also examined the vaccination rates between men and women. The results showed that women (71.2%) had a significantly (Χ2 = 11.8; p < 0.05; n = 115) higher immunity to varicella than men (39.7%). Furthermore, there were no significant differences between the vaccination rates of women and men, but a weak correlation between the type of transplantation and vaccination rate as well as sex and vaccination rate (Kendall tau b value < 0.3, respectively).

Last, we compared the vaccination rates for indicated vaccinations before and after transplantation by conducting Wilcoxon test (Table 4). This showed significantly lower vaccination rates for HepA and B as well as for COVID-19 (HepA: 37% vs. 16%, z = -3.479, p < 0.010; HepB: 62% vs. 21%, z = -5.250, p < 0.010; COVID-19: 97% vs. 19%, z = -5.000, p < 0.010). There was a trend towards higher influenza vaccination rates after transplantation, although not statistically significant (52% vs. 64%, z = -1.809, p = 0.070).

**Table 4:** Participants (n) who received at least one indicated vaccination before and after transplantation.

	At least one vaccination pre-transplantation (n/%)	At least one vaccination post-transplantation (n/%)	Wilcoxon test, z-score	Significance p
HepA*	38/37%	16/16%	-3.479	< 0.010
HepB*	64/62%	22/21%	-5.250	< 0.010
MenACWY*	7/7%	3/3%	-1.265	0.206
MenB*	3/3%	5/5%	-0.707	0.480
PCV*	33/32%	34/33%	-0.137	0.891
Influenza*	54/52%	66/64%	-1.809	0.070
COVID-19**	31/97%	6/19%	-5.000	< 0.010

Results of performed Wilcoxon test to determine any differences in vaccination rates are presented below.

*Participants transplanted at least data collection (n = 103).

**Participants transplanted at least data collection, but after 01.01.2021, when COVID-19 vaccine became available (n = 32).

### Evaluation of vaccination motivation

**Fig. 2:**
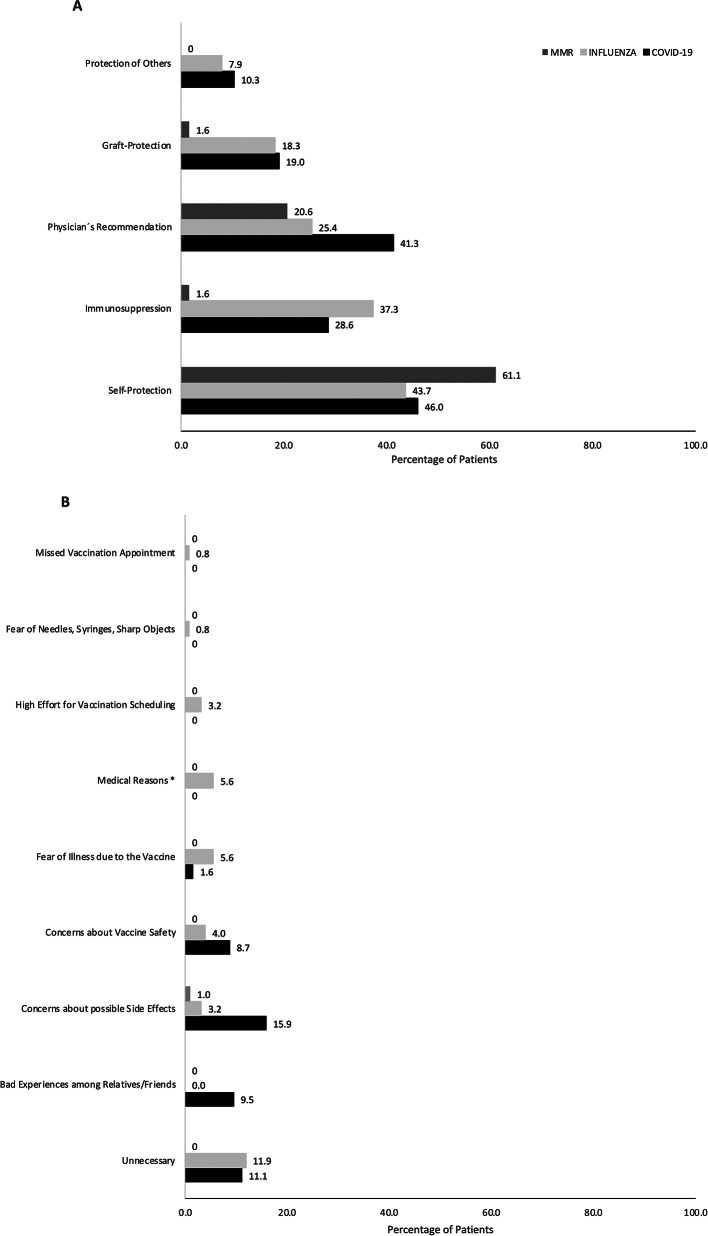
Personal reasons for vaccination (**A**) and Personal reasons against vaccination (**B**) against coronavirus COVID-19, measles, mumps, and rubella (MMR), and Influenza, multiple selections possible (n = 126). The primary rationale for vaccination is the individual’s wish to protect themselves, the physician’s recommendation, and individual awareness of their immunosuppression. The most common reasons against vaccinations are potential adverse effects and not necessary. *No rational contraindication (such as dermatoses, indisposition, etc.)

The primary rationale for vaccination is the individual’s wish to protect themselves. Other significant factors influencing vaccination decisions include recommendations from treating physicians and the individual’s awareness of their immunosuppression. The most common reasons for declining vaccination were concerns about potential adverse effects and the perception that vaccination was unnecessary. Figure 2 shows an overview of pro and contra as well as the resulting vaccination motivation.

A Spearman-Rho correlation was used for the statistical evaluation of vaccination motivation. According to the Likert scale results, no significant correlation between knowledge about vaccinations or information about recommended vaccinations and trust in vaccinations or future willingness to be vaccinated was found. A comparison of men and women showed that men had significantly higher confidence in vaccinations than women (U = 1492; Z = − 2.279; p < 0.05). In addition, the willingness to be vaccinated had changed significantly at a higher rate in men than in women since transplantation (U = 1425; Z = − 2.921; p < 0.05).

## Discussion

The present study addresses an existing research gap, as vaccination rates play a pivotal role in the success of organ transplantation. Until now, vaccination status has only been recorded for certain indicated vaccinations^14–16,18–24^, but not for all recommended vaccinations for transplant patients. However, the recommendations of the Robert Koch Institute do not differentiate between the individual types of transplantation^15^. It also recommends that vaccinations be administered and completed before transplantation^15,25^. Our procedure allows us a comparative analysis across different transplant types. This approach enables the identification of similarities and differences that contribute to maintaining an up-to-date vaccination status.

Furthermore, only patients after transplantation were included in this study, therefore comparing pre- or post-transplant vaccinations was feasible. Given the ongoing public debate on vaccinations, it is possible that individuals with more skeptical attitudes declined to participate.

The vaccination status can be determined in various ways, including databases or electronic patient records of health authorities or transplant centers^14,20^, serological immune status testing^14,19^, or vaccination cards^23,24^. The latter offers a quick and easy data collection, without further blood samples or waiting times for the patient. However, the vaccination card only provides information on the vaccinations carried out and documented, not the actual immunization status. Unfortunately, the serological response in transplant patients is often reduced compared to immunocompetent patients^14,18^. Consequently, it may be necessary to vaccinate these patients more frequently. Serological testing in transplant patients would certainly be useful, but is not available for all recommended vaccinations.

Numerous US studies are based on the guidelines of the American Society of Transplantation Infectious Disease Community of Practice^14,21,25^. One study of transplant patients found vaccination rates of 57–63% for influenza, and 56% for PCV, with no influence of sex and social status, but lower rates in rural areas. Liver and lung recipients were the least likely to be vaccinated against influenza^21^. An additional standardized serological screening procedure for MMR, VZV and other infectious diseases could be essential not only for enhancing vaccination, but for immunization rates^14^. In Germany, the last known survey of the vaccination status of transplant patients was collected in 2014 and published in 2016. However, only liver transplant patients and selected vaccinations were analyzed^23^. In our study, liver, kidney, and pancreas transplants were included analyzing standard, indicated and risk-dependent vaccination status.

The HepB vaccination rate of kidney transplant patients (88.1%) is significantly higher in our study than that of liver transplant patients, 55.4%. This is consistent with the data from the literature^26^. The reason for this is the recommendation of a HepB vaccination already during hemodialysis and thus before transplantation^27^. By contrast, vaccination rates for Hep A, Hep B, and COVID-19 were significantly lower after transplantation. Despite the safety of hepatitis post-transplantation vaccinations, the vaccination rate remains suboptimal^28^. Comprehensive recommendations for follow-up vaccination for primary care physicians have led to a significant improvement in vaccination rates before liver transplantation^29^ and could be adapted in the post-transplant setting. Furthermore, there was a significantly higher immunity to varicella, with women also showing a higher immunity (71.2% vs. 39.7%). The observed higher immunity to varicella among women than men may be attributed to targeted vaccination efforts before pregnancy, as varicella infection poses significant risks during pregnancy. There is no comparable evidence for this in the literature. Moreover, the analysis suggested a non-significant trend toward higher influenza vaccination rates post-transplantation. The rates align with the existing literature on influenza vaccination rates in transplant patients^21^.

Beyond this, no sex-specific differences were found, a finding that aligns with previous observations^21^. Therefore, strategies to improve vaccination rates need to be developed unrelated to sex, social status and transplant type. With increasing age, awareness of the need for vaccinations appears to increase, but differs between urban and rural areas^21^.

This study combines a survey of vaccination coverage with an assessment of vaccination motivation. The aim was to identify possible determinants influencing patients’ willingness to be vaccinated. In the past, questionnaires have been used to assess the willingness of kidney transplant patients to be vaccinated against influenza or pneumococcal infections (PCV)^22^. Factors that negatively influence the vaccination rate are lack of reimbursement by health insurance companies, age-related restrictions on vaccination recommendations, lack of availability of vaccinations at the time of evaluation, or lack of commitment to vaccination recommendations^30^. Most of the patients questioned in the survey reported that they were willing to take the vaccinations recommended by their treating physicians and could openly discuss their concerns about vaccinations with their physicians. A review discussed that the attitude and recommendations of the treating physicians have a major influence on the vaccination of vulnerable patient groups, such as immunosuppressed patients^31^. The higher the level of trust in vaccinations, the lower the patients’ assessment of the risk of side effects. Anxiety about side effects is an essential factor in patients’ willingness to be vaccinated. Promoting their confidence positively influences their desire to be vaccinated^32^. As shown in our study, confidence in vaccinations correlates negatively with the fear of side effects from, e.g., influenza vaccination or MMR vaccination.

A consistent assessment of vaccination status pre- and post-transplantation, a detailed discussion on infection prevention measures, and the direct offering of vaccinations in outpatient clinics are essential steps. These consultations can also address patients’ personal attitudes, helping to build trust and reduce concerns about side effects, improving motivation for vaccination. Electronic reminders for patients and primary care physicians can further support vaccination adherence^33^. In other European countries, like Denmark and France, various strategies have already been scientifically studied^34,35^. Including the involvement of clinical pharmacists in patient care could serve as potential models. Their role in patient education, medication management, and vaccination counseling has shown promising results in improving vaccination rates. Implementing similar approaches in transplant care may help optimize vaccination motivation.

Physicians play a crucial role in further improving transplant patients’ infection protection. It is thus necessary to train them regularly about the recommended vaccinations for this patient group supported by pharmacists.

This study has several limitations. Firstly, it can be assumed that people with a critical and skeptical opinion of vaccinations declined to participate in the study. All patients were offered participation in the study (by telephone or directly) during the study period, 10.0% (n = 14) refused to participate. Our patient cohort might suggest a higher level of vaccination protection. Secondly, any “non-remember” responses were classified as not vaccinated. A more comprehensive set of data concerning physicians was not collected as part of this study, since the physicians treating the patients were not interviewed. Vaccination rates might have appeared higher if serological tests had been included. However, due to the additional effort, costs, and limited availability of titer tests for all recommended vaccinations, serology was not performed.

## Conclusion

Transplant patients are at an elevated risk for infections, making infection prevention crucial in their care. This study addresses a significant research gap by focusing on the vaccination status, which is vital for the success of organ transplantation. We employed two distinct approaches to assess vaccination status and motivation. First, we analyzed patients’ vaccination cards based on German vaccination recommendations, categorizing vaccinations into standard, indicated, and risk-dependent. Second, we evaluated vaccination motivation through semi-structured interviews using a self-developed questionnaire. Our findings reveal that only a few transplant patients have complete vaccination status. Enhanced efforts are necessary to boost patients’ confidence in vaccinations and motivate them. This study offers the most comprehensive overview to date of vaccination rates among transplant patients according to German vaccination guidelines.

## Data Availability

The datasets used and/or analysed during the current study available from the corresponding author on reasonable request.

## Declarations

### Statement on the procurement of organs/tissues

In accordance with the guidelines on human transplantation studies by Scientific Reports, we ensure that no organs/tissues were procured from prisoners. The study examined patients who underwent transplantation in Germany in accordance with the German Transplantation Act (TPG).
